# KDM4B facilitates colorectal cancer growth and glucose metabolism by stimulating TRAF6-mediated AKT activation

**DOI:** 10.1186/s13046-020-1522-3

**Published:** 2020-01-13

**Authors:** Haijie Li, Jingqin Lan, Guihua Wang, Kaixuan Guo, Caishun Han, Xiaolan Li, Junbo Hu, Zhixin Cao, Xuelai Luo

**Affiliations:** Department of Gastrointestinal Cancer Research Institute, Tongji Hospital, Tongji Medical College, Huazhong University of Science and Technology, Wuhan, Hubei China

**Keywords:** KDM4B, GLUT1, AKT activation, TRAF6, Glucose metabolism

## Abstract

**Background:**

Histone lysine demethylase 4B (KDM4B) has been implicated in various pathological processes and human diseases. Glucose metabolism is the main pattern of energy supply in cells and its dysfunction is closely related to tumorigenesis. Recent study shows that KDM4B protects against obesity and metabolic dysfunction. We realized the significant role of KDM4B in metabolism. However, the role of KDM4B in glucose metabolism remains unclear. Here, we sought to delineate the role and mechanism of KDM4B in glucose metabolism in colorectal cancer (CRC).

**Methods:**

We first analyzed the role of KDM4B in glucose uptake and CRC growth. We then investigated the consequences of KDM4B inhibition on the expression of GLUT1 and AKT signaling, also explored the underlying mechanism. Finally, we detected the mechanism in vivo and assessed the potential correlation between the expression of KDM4B and CRC prognosis.

**Results:**

We found that KDM4B promoted glucose uptake and ATP production by regulating the expression of GLUT1 via the AKT signaling pathway. KDM4B could interact with TRAF6 and promote TRAF6-mediated ubiquitination of AKT for AKT activation. Furthermore, we demonstrated that KDM4B was overexpressed in CRC specimens and high level of KDM4B was associated with a poor survival rate in CRC patients.

**Conclusions:**

These findings reveal that KDM4B plays an important role in promoting CRC progression by enhancing glucose metabolism.

## Background

Colorectal cancer (CRC) is one of the most common cancers in the world. It is the third most commonly diagnosed cancer in men and the second in women worldwide [1]. In Asian countries, the incidence of CRC has been rising rapidly [2]. However, the underlying molecular mechanisms responsible for CRC tumorigenesis are still not fully understood.

The metabolic properties of cancer cells are distinct from those of normal cells [3].The best characterized metabolic phenotype observed in tumor cells is the Warburg effect, which is a shift from ATP generation through oxidative phosphorylation to ATP generation through glycolysis, even in the presence of sufficient oxygen [4]. Although glycolysis can make ATP production more rapidly than oxidative phosphorylation, it is far less efficient in terms of ATP generated per unit of glucose consumed. This shift therefore demands that tumor cells implement an abnormally high rate of glucose uptake to meet the increased energy needs [5]. In the regulation of glucose uptake, the PI3K/AKT signaling plays a role of master regulator. PI3K/AKT signaling promotes both the expression of glucose transporter GLUT1 and the translocation of GLUT1 protein from the endo-membranes to the cell surface [6]. In addition, AKT potentiates the activity of the HK (Hexokinase), which phosphorylates glucose molecules, thus preventing their efflux back into the extracellular space [7].

KDM4B is an important member of the histone demethylase KDM4 family that is characterized by the catalytic Jumonji C domain. KDM4B is thought to regulate gene expression by demethylating tri-and di-methylated histone H3 at lysine 9 and lysine 36 [8]. Expression levels of KDM4B are notably upregulated in numerous cancers, including breast, prostate, bladder, ovarian, gastric and colorectal cancer [9–12]. It has been reported that KDM4B has a crucial role in human carcinogenesis by regulating cell cycle progression and apoptosis [13, 14]. KDM4B also plays an important role in cancer metastasis, DNA damage response and cell differentiation [12, 15, 16]. Recent study shows that KDM4B protects against obesity and metabolic dysfunction [17]. It is made us realized the significant role of KDM4B in metabolism. However, little is currently known about the role of KDM4B in glucose metabolism.

In the present study, we investigated the role of KDM4B in glucose metabolism. Our results showed that KDM4B promoted glucose uptake and ATP production by regulating the expression of GLUT1 via the AKT signaling pathway through the combination with TRAF6. Furthermore, we demonstrated that KDM4B was overexpressed in CRC specimens and high level of KDM4B was associated with a poor survival rate in CRC patients. Collectively, we reveal that KDM4B plays an important role in promoting CRC progression by enhancing glucose metabolism.

## Materials and methods

### Cell lines and cell culture

The human CRC cell lines LoVo, SW620, HCT116 and human embryonic kidney HEK293 T were purchased from the Cell Bank of Type Culture Collection of Chinese Academy of Sciences (Shanghai, China). LoVo, SW620 and 293 T cells were cultured at 37 °C, 5% CO_2_ in DMEM medium containing 10% fetal bovine serum (GIBICO, NY, USA). HCT116 cells were cultured in McCoy’s 5A medium containing 10% fetal bovine serum (GIBICO).

### Antibodies

The following antibodies were used for Western blot analysis, Co-IP, immunofluorescence or immunohistochemistry assay: anti-KDM4B (2639, Cell Signaling Technology), anti-GLUT1(12,939, Cell Signaling Technology), anti-AKT (2920, Cell Signaling Technology), anti-Phosoho-AKT Ser473 (4060, Cell Signaling Technology), Phosoho-AKT Thr308 (4056, Cell Signaling Technology), anti-TRAF6 (8028, Cell Signaling Technology), anti-6X His tag(ab18184, Abcam), anti-HA tag(ab9110, Abcam), anti-Flag tag(SAB4301135, Sigma-Aldrich), anti-TXNIP(14,715, Cell Signaling Technology).

### RNA interference and expression plasmid

siRNA duplexes targeting the human *KDM4B* gene were synthesized and purified by RiboBio (Ribobio, Guangzhou, China). siRNA duplexes with non-specific sequences were used as an siRNA-negative control. RNA oligonucleotides were transfected using Lipofectamine RNAiMAX Reagent (Invitrogen) and the expression levels of KDM4B were quantified 72 h after transfection. KDM4B siRNA was designed as follows: siKDM4B 1# 5′- GCGCAGAAUCUACCAACUU-3′, siKDM4B 2# 5′- CGGCCACAUUACCCUCCAA-3′.

cDNA constructs encoding KDM4B were cloned into the pcDNA3.1 expression vector and Flag expression vector using standard cloning methodology. TRAF6 with HA-tag and AKT with HA-tag was also constructed by our team in the same way. Ubiquitin with his-tag was bought from Biovector Science Lab (NTCC, Beijing, China). The myr-AKT plasmid was a generous gift from Dr. Hui Kuan Lin (Department of Cancer Biology, Wake Forest Baptist Medical Center, NC, USA).

Eukaryotic expression plasmids (1μg) were transfected into 293 T cells or CRC cells in 6-well plates using 10 ul Lipofectamine 3000 (Invitrogen). Cells were harvested after 72 h for further analysis.

### Cell cycle analysis and BrdUrd incorporation analysis

Cells were fixed in 80% ethanol overnight at − 20 °C, washed with phosphate-buffered saline, and then stained with propidium iodide and 100μg/ml RNaseA. DNA content was measured by sorting the fluorescence-activated cells on a Becton-Dickinson FACScan System (Franklin Lakes, NJ, USA). For the BrdUrd incorporation analysis, cells were incubated in BrdUrd medium at 10μg/ml for 30-min. After aspirating the medium, the cells were immediately fixed for more than 8 h at − 20 °C. After immunostaining using the BrdUrd antibody, the DNA synthesis rate was assessed by calculating the percentage of BrdUrd^+^ cells out of the total cell count on BD FACScan System.

### Glucose uptake and ATP detection

Intracellular glucose uptake was determined by 2-deoxyglucose-6-phosphate (2DG6P), a fluorescently-tagged glucose derivative, using a Glucose Uptake Cell-Based Assay Kit (Promega, WI, USA) in accordance with the manufacturer’s protocol. The cells were incubated with 2-deoxyglucose (2DG) for 10 min in 96-wells and the followed with the protocol to detect the luminescent signal that is proportional to the concentration of 2DG6P.

Cellular ATP levels were measured using a firefly luciferase-based Bioluminescence ATP assay kit (Beyotime, Jiangsu, China). Briefly, cells were lysed and centrifuged at 12,000 g for 5 min at 4 °C. Then 100ul of each supernatant was mixed with 100ul of ATP detection solution. Luminance (RLU) was measured by a Luminometer.

### Immunofluorescence

Cells were rinsed with PBS, fixed in 4% paraformaldehyde for 10 min at room temperature, and permeabilized with 0.1% Triton X-100 for 10 min. The cells were blocked with 2% BSA-PBS at room temperature for 60 min, and incubated with primary antibodies at 4 °C overnight. After washing 3 times with PBS, cells were incubated with secondary antibodies for 60 min at 37 °C, and fluorescence at 488 nm (FITC) and 543 nm (TRITC) were detected using the LSM 710 confocal laser scanning microscope.

### Cell fractionation

Cytosolic and membrane fractions were prepared using the Mem-PER Plus Membrane Protein Extraction Kit (Thermo Fisher) according to the manufacturers’ standard procedures.

Briefly, cells were suspended in Permeabilization Buffer for 10 min at 4 °C. The permeabilized cells were centrifuged at 16000 g for 15 min at 4 °C. The resulting supernatant (cytosol) was collected and the sediment was resuspended by Solubilization Buffer. The lysate was again centrifuged at 16000 g for 15 min at 4 °C and the supernatant (membrane) was collected. The distribution of proteins in the cytosol and membrane fractions was analyzed by western blotting.

### Immunoprecipitation and immunoblotting

Two hundred ninety-three T cells were cultured in DMEM medium containing 10% FBS. Transfection was performed with Lipofectamine 3000 and RNAiMAX (Invitrogen, California, USA). At 24-48 h after transfection, cells were harvested and lysed in NP40 lysis buffer supplemented with protease inhibitor cocktail (Beyotime, Jiangsu, China). Immunoprecipitation and immunoblotting were performed as described in Luo Zhang’s article [18]. The Gel-pro Analyzer 4.0 was used to quantify the bands in immunoblotting images.

To detect endogenous interaction between KDM4B and TRAF6, immunoprecipitation was performed with 2μg of agarose-conjugated anti-TRAF6 antibody. Cells were incubated with IGF-1(100 ng/ml) for 0, 15, 30 and 60 min and then collected for the immunoprecipitation.

### Lentiviral system

The generation of stable expression cells was performed by lentiviral system. KDM4B was depleted in LoVo and HCT116 cells using a KDM4B lentiviral construct expressing KDM4B-targeted short hairpin RNA (shKDM4B, Genechem, Shanghai, China). Following infection with lentivirus, cells were expanded in medium containing puromycin (1.5 μg/ml), and screened for KDM4B knockdown by western blot analysis. Empty vector-infected cells (shControl) were used as a control. Myr-AKT stable overexpression cells and control cells were generated in the same way.

### In vivo tumorigenesis

The study was approved by the Huazhong University of Science and Technology (HUST) Ethics Committee. All animal experiments were performed accordance with the animal study guidelines of HUST. Nude mice (nu/nu, female, 5 weeks old) were injected subcutaneously with CRC cells (1 × 10^6^ cells) stably expressing the control and shcontrol vector (shControl+Control group), myr-AKT with shcontrol vector (shControl +AKT group), shKDM4B with control vector (shKDM4B + Control group) and shKDM4B with myr-AKT vector (shKDM4B + AKT group). Ten animals per group were used in each group. Tumor growth was monitored for 35 days. Tumor size was measured with a caliper, and tumor volume was determined with a standard formula, L × W^2^ × 0.5, where L is the longest diameter and W is the shortest diameter. The tumors were dissected and analyzed every 5 days. Mice were sacrificed by cervical dislocated at indicated time and the tumors were removed for analysis.

### Tissue microarray and immunohistochemistry

A commercially available paraffin-embedded human CRC tissue array was bought form Shanghai Outdo Biotech, catalog no. HColA180Su10. Demographic and clinicopathological data including clinical staging (According to the AJCC staging system) and survival data were provided by the manufacturer. Immunohistochemistry was performed as described previously [9].

KDM4B immunostaining was evaluated based on scores representing the percentage of positively stained tumor cells and the staining intensity grade. KDM4B positive cells were counted in 3 different fields and photographed using an Olympus microscope. The immunoreactions were evaluated independently by two pathologists blinded to the clinicopathologic information to ensure proper tissue morphology. The percentages of positively stained tumor cells were scored according to the following scale: 0 (no positive cells), 1 (< 25% positive cells), 2 (25–50% positive cells), 3 (50–75% positive cells), and 4(> 75% positive cells). The staining intensities were classified into the following four categories: 0 (no staining); 1 (weak staining); 2 (moderate staining), 3 (strong staining). The score for each tissue was calculated by multiplying the intensity index with the percentage scale. The median value of KDM4B scores was employed to determine the cutoff. Tumors with KDM4B scores lower or equal to the median were designated as “low expression”, whereas those with scores higher than the median were designated as “high expression”.

### Statistical analysis

The results were analyzed using SPSS 19 (Chicago, IL, USA). The data were expressed at the mean ± SD. KDM4B expression between tumor tissues and matched nontumor tissues was analyzed with the paired Student’s t test. The association between KDM4B expression and various clinicopathological parameters was evaluated with the χ^2^ test. The Cox proportional hazard regression model was used for univariate and multivariate analyses to determine the effects of the clinicopathological variables and KDM4B expression on the patient survival. Only variables with *p* values < 0.05 in univariate analysis were included in multivariate analysis. Survival curves were calculated using the Kaplan-Meier method. In all statistical tests, p values < 0.05 were deemed statistically significant.

## Results

### KDM4B is necessary for CRC cells proliferation and glucose metabolism

**Fig. 1:**
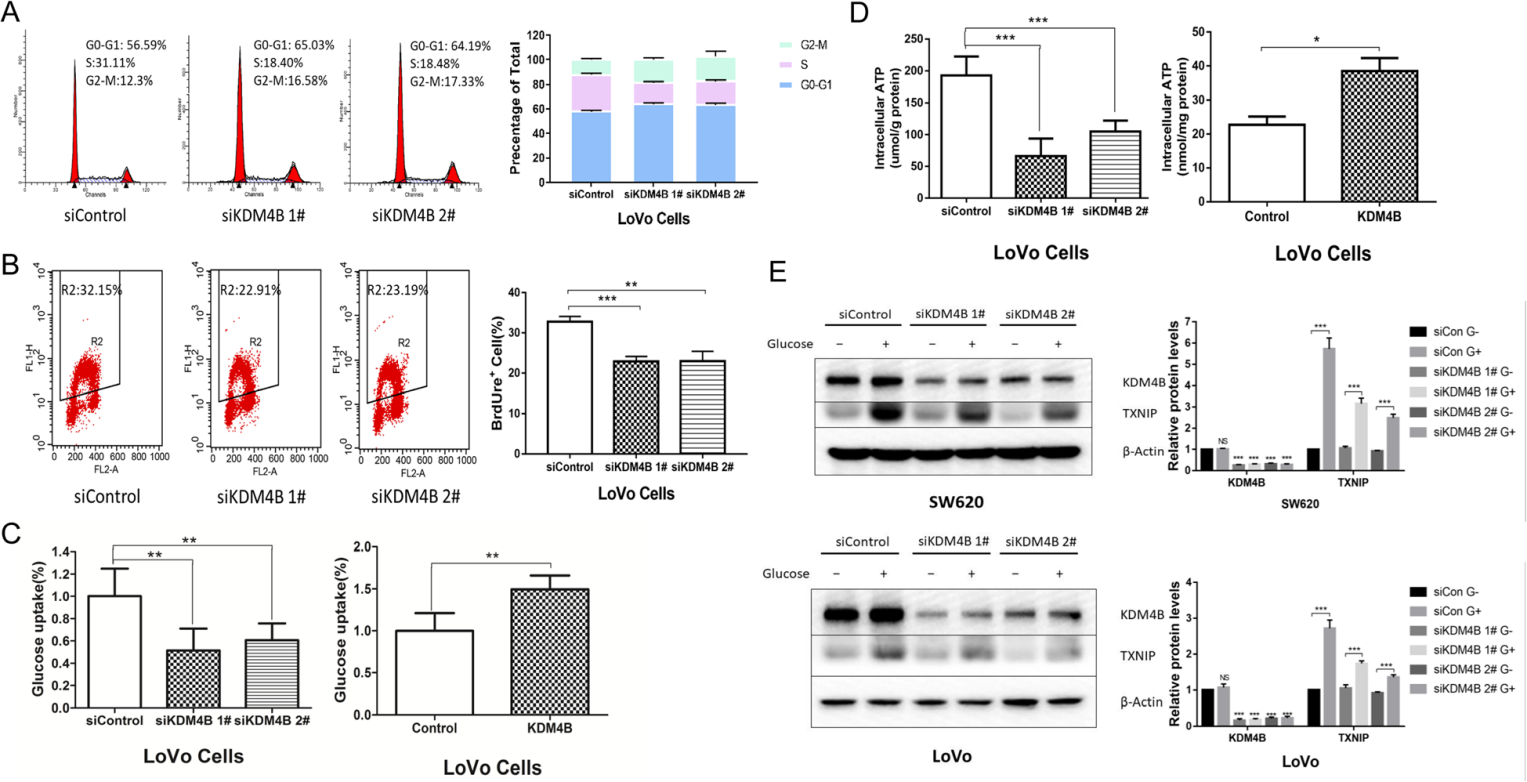
KDM4B is necessary for CRC cells proliferation and glucose metabolism. **a** Cell-cycle progression analysis was measured by propodium iodide staining and flow cytometry in LoVo cells transfected with siControl and siKDM4B 1#/2#. Representative histograms from an individual experiment and similar results were obtained in three independent experiments. **b** BrdUrd incorporation into DNA and DNA content in nuclei were determined by flow cytometry analysis in LoVo siControl and siKDM4B 1#/2# cells. **c** Intracellular glucose uptake was evaluated by 2-NBDG, a fluorescently tagged glucose derivative in KDM4B-depressed LoVo cells (siKDM4B 1#/2#) and KDM4B-overexpressed LoVo cells (KDM4B). **d** Intracellular ATP levels were measured by firefly luciferase-based bioluminescence ATP assay in KDM4B-depressed/overexpressed LoVo cells. **e** The expression of glucose-induced TXNIP was used to sense intracellular glucose uptake. Cells were incubated in glucose-free medium for 12 h, followed by glucose stimulation for an additional 3 h in LoVo cells and SW620 cells

Initially, we performed functional analysis using CRC cell lines to elucidate whether KDM4B was involved in proliferation and affected glucose metabolism in CRC. To identify the roles of KDM4B in cell proliferation, we detected the cell-cycle progression and DNA synthesis in KDM4B-depressed cells. We found that KDM4B knockdown caused a significant decrease in the cell number at S-phase and incorporated BrdUrd, showing that the knockdown of KDM4B blocked the cell-cycle procession and inhibited the DNA synthesis (Fig. 1a and b). In the meantime, KDM4B overexpression promoted the DNA synthesis (Additional file 1: Figure S1A).

In order to fulfill the biosynthetic demands associated with rapid proliferation, cancer cells must increase the import of nutrients form the environment. The main nutrient that supports survival and biosynthesis in mammalian cells is glucose. So we detected the role of KDM4B in glucose uptake which was the initial step of glucose metabolism. Our results showed that intracellular glucose uptake was significantly decreased in KDM4B-depressed cells and increased in KDM4B-overexpressed cells (Fig. 1c and Additional file 1: Figure S1B). Furthermore, KDM4B-depressed cells displayed a significant decrease of cellular ATP content while KDM4B-overexpressed cells displayed a significant increase (Fig. 1d). We also examined glucose-induced TXNIP expression, which is commonly used as an intracellular glucose sensor [19, 20]. TXNIP was robustly induced in control cells following glucose stimulation. In contrast, the induction of TXNIP was suppressed in KDM4B-depressed cells, supporting the result that the depression of KDM4B inhibited intracellular glucose uptake in CRC cells (Fig. 1e). To further explore potential functions of KDM4B in CRC, gene expression profiling on GSE9348 from GEO database was employed. CRC tissues had more abnormal genes expression in Glucose transport pathway than normal tissues (Additional file 1: Figure S1C).

Overall, these results of functional analysis suggested that KDM4B promoted the proliferation and glucose metabolism of CRC cells through regulation glucose uptake, which encouraged us to further explore the significance of KDM4B in CRC metabolism.

### KDM4B promotes the expression of GLUT1 via the AKT signaling pathway

Glucose uptake is controlled primarily by the glucose transporter family (GLUTs) which have 14 members [21]. GLUT1, the first member of the GLUT1 family to be identified, has been the most extensively studied. GLUT1 is overexpressed in many tumors, including hepatic, pancreatic, breast, colorectal and ovarian cancers [22–25]. GLUT1 is the main glucose transporter in most cell types, and unlike GLUT4, it appears to be regulated primarily through alterations in expression levels [26]. Since GLUT1 is essential for cancer cell glucose uptake, we then examined the effect of KDM4B on GLUT1 expression. As shown in Additional file 1: Figure S2 A-E, the expression of GLUT1 was suppressed in KDM4B-depressed cells and was upregulated in KDM4B-overexpressed cells. These data indicate that KDM4B is involved in the regulation of GLUT1 expression.

**Fig. 2:**
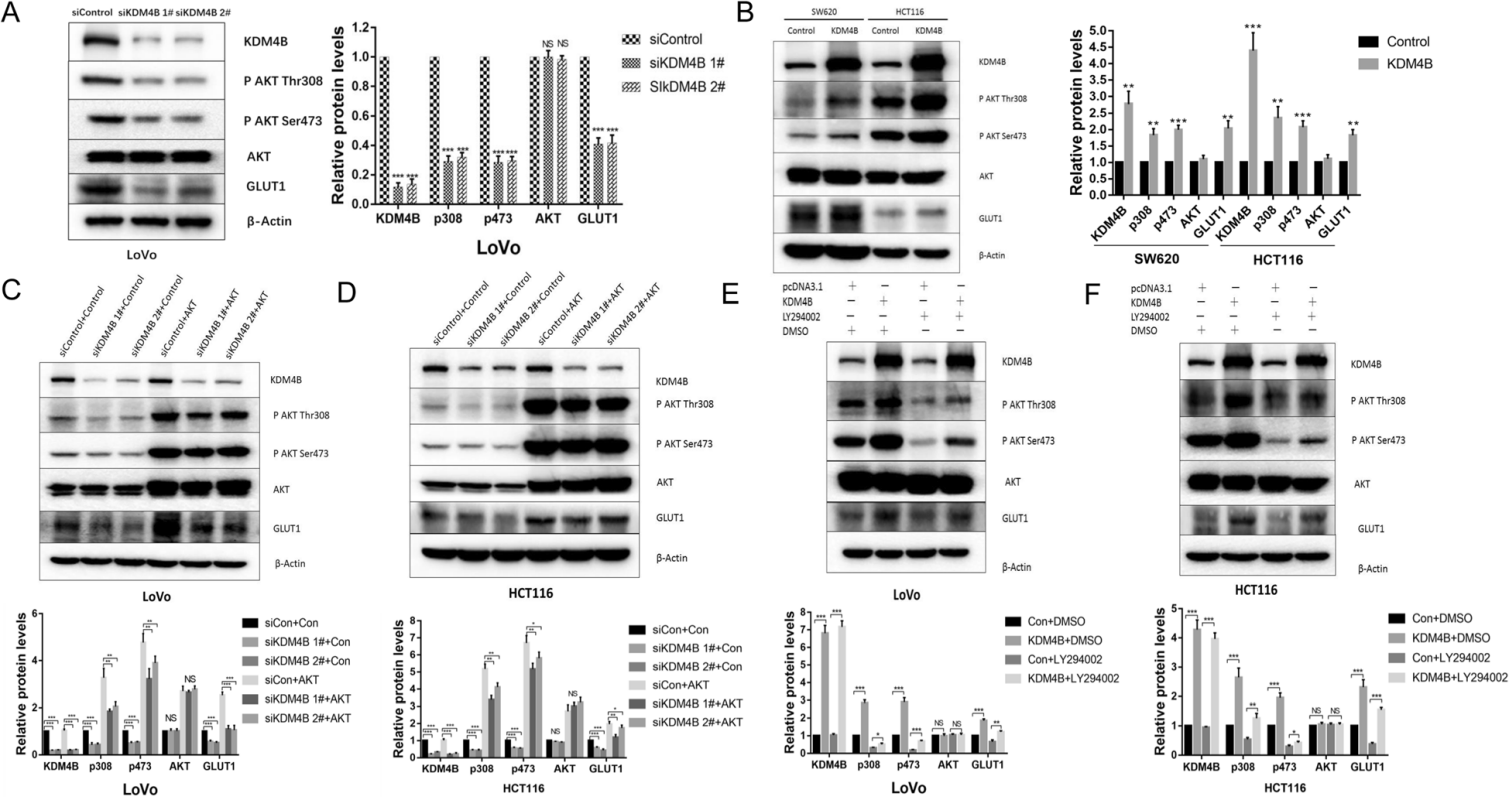
KDM4B promotes the expression of GLUT1 via the AKT signaling pathway. **a** and **b** The phosphorylation of AKT at Thr 308 and Ser 473 and the expression of GLUT1 was detected in KDM4B-depressed(A)/overexpressed(B) CRC cells. **c** and **d** The expression of GLUT1 was detected in KDM4B-depressed LoVo/HCT116 cells transfected with myr-AKT. **e** and **f** The expression of GLUT1 was detected in KDM4B-overexpressed LoVo/HCT116 cells incubated with PI3K inhibitor LY294002

In the regulation of GLUT1, the PI3K/AKT signaling plays a role of master regulator. To clarify the mechanism by which KDM4B regulate GLUT1, we further explored the role of KDM4B in AKT signaling pathways. The phosphorylation of AKT at Thr308 and Ser473 was inhibited in the KDM4B-depressed cells with the depressed GLUT1 (Fig. 2a and Additional file 1: Figure S3 A). Similarly, the phosphorylation of AKT at Thr308 and Ser473 was enhanced in KDM4B-overexpressed cells with the upregulated GLUT1 (Fig. 2b). To further test whether the regulation of GLUT1 by KDM4B depends on AKT or not, we enhanced the activation of AKT in KDM4B-depressed cells by transfected with a constitutively active mutant of AKT, myristoylated AKT (myr-AKT) which does not require its PH domain for plasma membrane recruitment, to detect the expression of GLUT1. We observed sustained phosphorylation of AKT efficiently rescued the expression of GLUT1 in KDM4B-depressed cells (Figure 2c and d). Similarly, the KDM4B-overexpressed cells with the PI3K inhibitor LY294002 efficiently reduced the expression of GLUT1(Fig. 2e and f). These data suggest that the regulation of GLUT1 by KDM4B depends, at least partially, on AKT.

### KDM4B promotes membrane localization of AKT

**Fig. 3:**
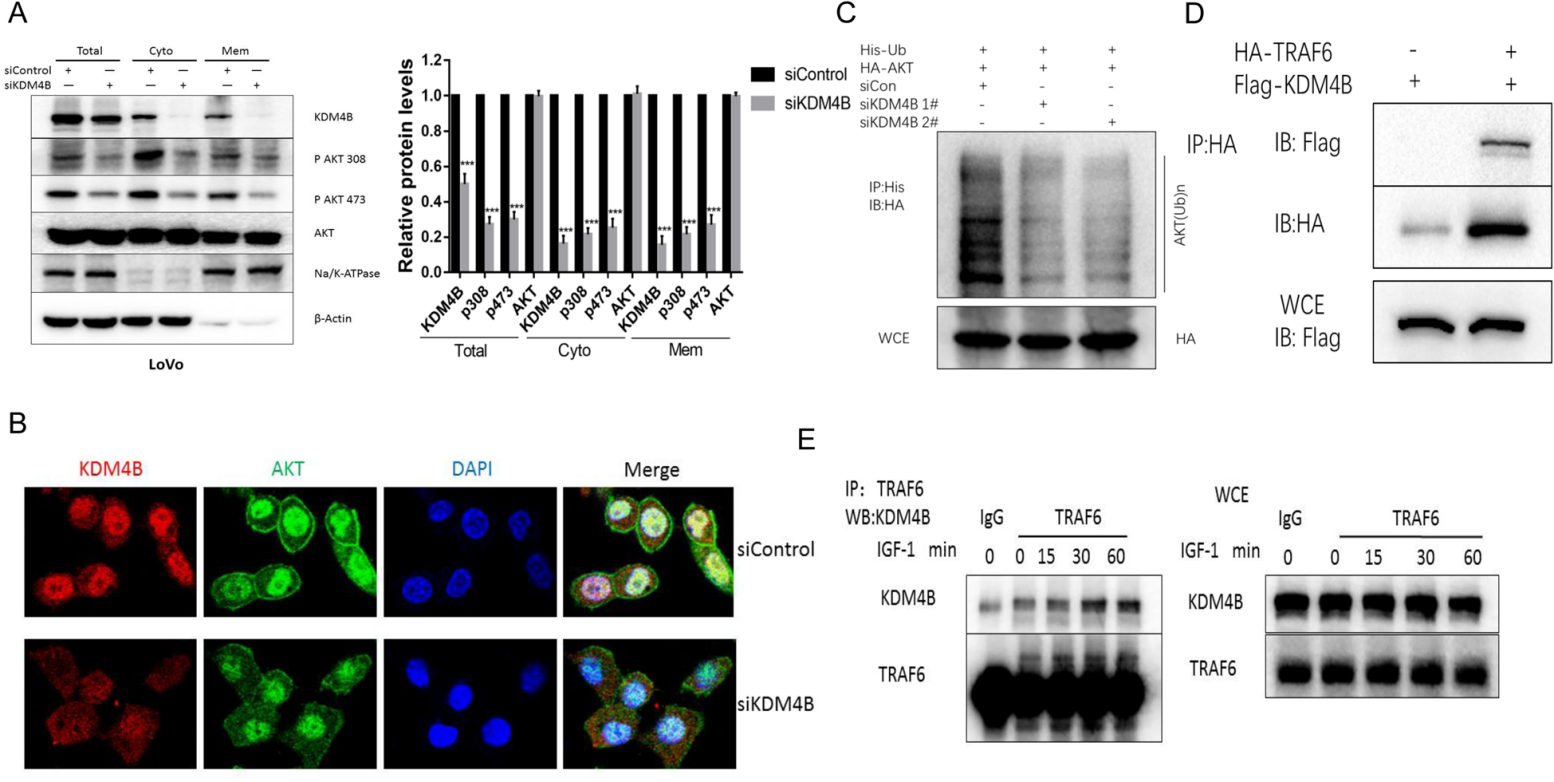
KDM4B promotes the membrane localization of AKT by promoting TRAF6-mediated ubiquitination of AKT. **a** The membrane fractions, cytoplasm fractions and whole cell extracts were collected in KDM4B-depressed LoVo cells to measure the phosphorylation of AKT at Thr308 and Ser 473. **b** KDM4B-depressed LoVo cells and control cells were fixed for immunofluorescence analysis by confocal microscopy. **c** In vivo ubiquitination assay in KDM4B-depressed 293 T cells transfected with HA-AKT, His-UB. Ubiquitinated AKT was detected in AKT immunoprecipitates. **d** HA-TRAF6 and Flag-KDM4B were transfected into 293 T cells as indicated. Flag-KDM4B proteins were immunoprecipitated by anti-HA antibody. Both the whole-cell lysates (WCE) and immunoprecipitated were analyzed by IB with anti-HA or anti-Flag antibody. **e** LoVo cells were stimulated with IGF-1 for the indicated times. The cell lysates were immunoprecipitated with anti-TRAF6 antibody or control IgG. Both the WCE and the immunoprecipitates were analyzed to detect the expression of KDM4B and TRAF6

AKT is generally activated by stimulation of growth factor receptors on the surface in a multistep process that includes binding of AKT to PIP3, translocation of AKT from the cytosol to the membrane and phosphorylation of AKT at Thr308 and Ser473 by the upstream kinases PDK1 and mTORC2 [27, 28]. Consider that the binding to PIP3 and membrane translocation is the initial and essential step for AKT activation, we reasoned that a possible mechanism of KDM4B in AKT activation could be to promote the translocation of AKT from the cytosol to the plasma membrane. Our results showed that KDM4B knockdown inhibited the membrane phosphorylation of AKT (Fig. 3a and Additional file 1: Figure S3B). Consistently, immunofluorescence analysis showed that the localization of AKT proteins on the membranes were reduced in KDM4B knockdown cells than that of control cells (Fig. 3b). These results indicate that KDM4B promotes AKT activation through the regulation of AKT membrane recruitment.

### KDM4B interacts with TRAF6 and promotes TRAF6-mediated ubiquitination of AKT

Protein ubiquitination through Lys48(K48) of the ubiquitin chain generally targets proteins for degradation, whereas ubiquitination through K63 plays a critical role in signaling activation and protein trafficking [29, 30]. The K63 chain ubiquitination of AKT contributes to the enhancement of AKT membrane localization and phosphorylation [31]. As KDM4B promotes AKT membrane recruitment, we analyzed whether KDM4B regulated the ubiquitination of AKT. It has been proved that ubiquitination occurred through K63 but not through K48 in the absence of proteasome inhibitor MG132 [31]. The result showed that KDM4B knockdown inhibited the ubiquitination of AKT without MG132 (Fig. 3c).

It has been proved that the ubiquitin ligase TRAF6 promotes the K63-linked ubiquitination of AKT and is required for plasma membrane translocation of AKT [31]. As KDM4B promoted AKT membrane recruitment, we hypothesize that KDM4B may interact with TRAF6 to promote its effect on AKT. We observed that KDM4B interacted with TRAF6 upon IGF-1 stimulation (Fig. 3d and e). These results indicate that KDM4B interacts with TRAF6 and promotes TRAF6-mediated AKT activation.

### KDM4B promotes cells proliferation and glucose metabolism in a partly AKT-dependent manner

**Fig. 4:**
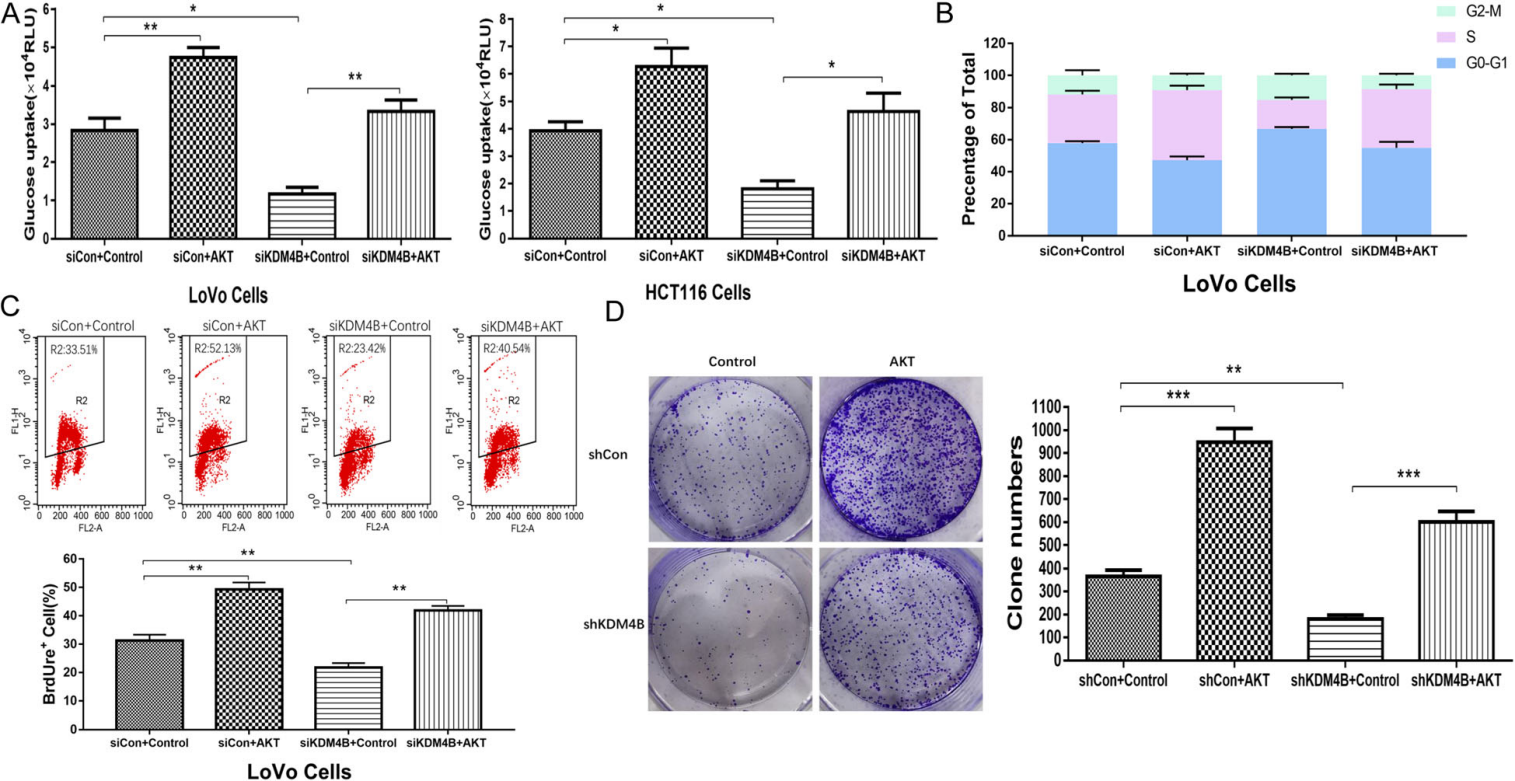
KDM4B promotes cells proliferation and glucose metabolism in a partly AKT-dependent manner. **a** Intracellular glucose uptake was evaluated by 2-NBDG in KDM4B-depressed LoVo/HCT116 cells transfected with/without myr-AKT. **b** Cell-cycle progression analysis was measured in KDM4B-depressed LoVo cells transfected with/without myr-AKT. **c** BrdUrd incorporation into DNA and DNA content in nuclei were determined by flow cytometry analysis in KDM4B-depressed LoVo cells transfected with/without myr-AKT. **d** Colony formation assay was performed in KDM4B-depressed LoVo cells transfected with/without myr-AKT

To identify potential role of AKT in the regulation of KDM4B in cells proliferation and glucose metabolism, we detected the functional implication including glucose uptake, cell-cycle progression, DNA synthesis and clone formation in KDM4B-depressed cells with enhanced AKT activity. We observed that the enhanced AKT activity can partly rescue the glucose uptake ability depressed by KDM4B knockdown (Fig. 4a). Similarly, enhanced AKT activity can partly rescue the cell-cycle procession (Fig. 4b), DNA synthesis (Fig. 4c) and clone formation (Fig. 4d) blocked by KDM4B knockdown. To make our results more convincing, we detected the effect of AKT inhibition on cells proliferation and glycometabolism in KDM4B-overexpressed cells. We found that AKT inhibition could partly depress the enhancement of DNA synthesis (Additional file 1: Figure S3 C) and glucose uptake (Additional file 1: Figure S3 D) by KDM4B-overexpressed. These results indicate that KDM4B promotes cells proliferation and glucose metabolism in a partly AKT-dependent manner.

### AKT inhibits KDM4B-depressed induction of tumor growth suppression in mouse xenograft models

**Fig. 5:**
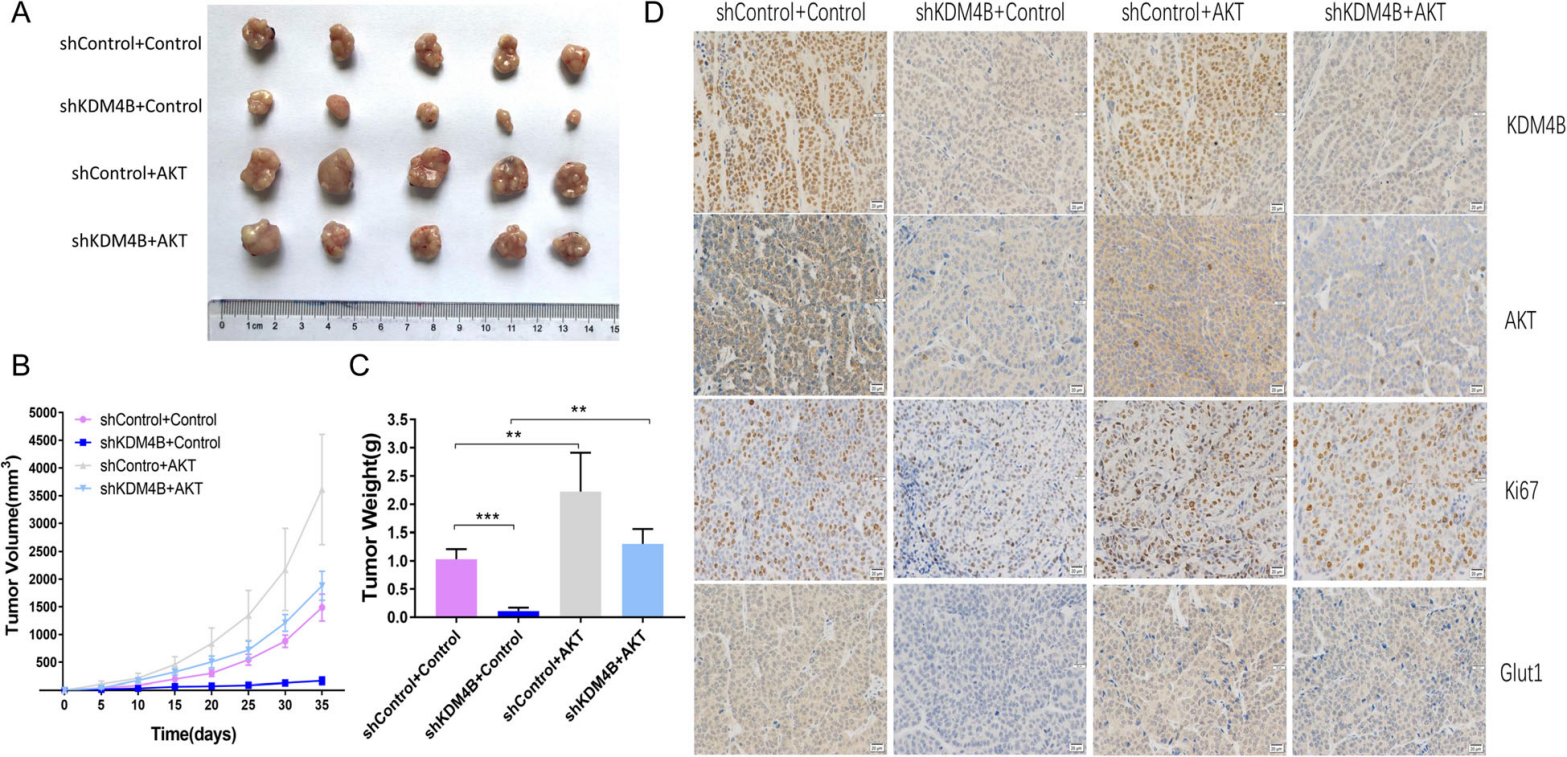
AKT inhibits KDM4B-depressed induction of tumor growth suppression in mouse xenograft models. **a** Stable KDM4B-depressed LoVo cells with/without constitutively active AKT and control cells were injected subcutaneously into nude mice. The tumors were collected and shown. **b** Tumor development was monitored for 35 days. The length and width of tumors were measured every 5 days to determine tumor volume. **c** The mean tumor weight of each group was calculated. **d** Representative images of immunohistochemical(IHC) staining (using anti-KDM4B/AKT/GLUT1/Ki67) of tumor tissues were shown

After confirming the mechanism that KDM4B promotes the expression of GLUT1 via the AKT signaling pathway in colorectal cells, we investigated whether AKT could rescue the KDM4B depress-induced suppression of tumor growth in mouse xenograft models. To test this, we generated colorectal cell lines expressing KDM4B shRNA, myr-AKT plasmid alone or simultaneously expressing KDM4B shRNA and myr-AKT plasmid by lentivirus. We found that KDM4B knockdown significantly suppressed tumor growth, both in the tumor volume and weight (Fig. 5a-c). But the inhibition of tumor growth by KDM4B knockdown was partially rescued when AKT was constitutively active in the meantime in vivo (Fig. 5a-c). In support of this, we detected lower Ki-67 staining in KDM4B knockdown tumor tissues and stronger Ki-67 staining in simultaneous KDM4B knockdown and AKT constitutively activation tumor tissues by the immunohistochemistry analysis (Fig. 5d). Collectively, these results demonstrate that the loss of KDM4B in CRC cells results in the suppression of tumor growth and the suppression can be partly rescued by constitutively active AKT.

### KDM4B is frequently up-regulated in colorectal tumor tissues and positive expression is correlated with an unfavorable prognosis

**Fig. 6:**
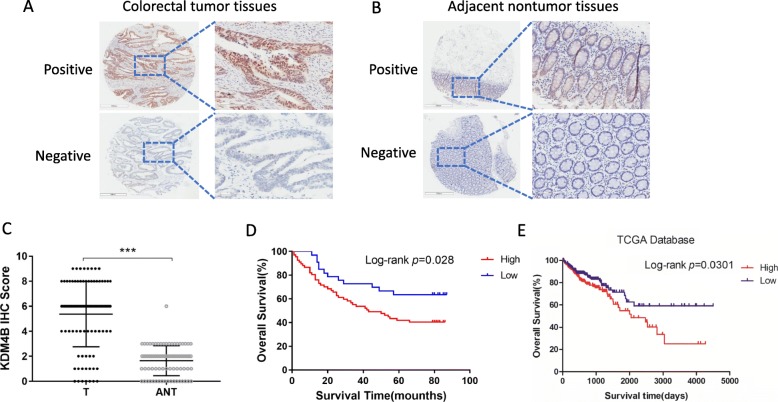
KDM4B is frequently up-regulated in colorectal tumor tissues and positive expression is correlated with an unfavorable prognosis. **a** and **b** Representative results of immunohistochemical staining for KDM4B expression in colorectal cancer tissues (**a**) and adjacent nontumor tissues (**b**). **c** Dot plots showing immunohistochemical scores and mean ± SD of KDM4B in colorectal tumor and NT tissues. **d** Kaplan-Meier survival curves of patients with colorectal cancer in the tissue microarray (*n* = 80) segregate into high- and low-KDM4B expression groups. **e** Kaplan-Meier survival curves of patients with colorectal cancer in TCGA database segregate into high- and low-KDM4B expression groups

Given the striking effect of KDM4B on tumor growth, we then questioned whether there was an association between KDM4B expression and clinical prognosis in patients with colorectal cancer. We next evaluated the expression of KDM4B by immunohistochemical staining on a tissue microarray containing 180 spots, of which 160 were paired CRC tissue and corresponding adjacent nonneoplastic mucosa tissue from 80 patients, and the remaining spots were CRC tissue form 20 other patients. Representative slides of CRC tissues and corresponding adjacent nonneoplastic mucosa tissue are shown in Fig. 6a and b. Notably,

the expression of KDM4B was up-regulated in tumor tissues than adjacent nonneoplastic mucosa tissue (Fig. 6c). Furthermore, we noted the existence of a significant correlation between tumor KDM4B expression levels and pathologic T stage, as well as clinical stage, who underwent pre-therapy surgery (Table 1). Univariate Cox regression analyses indicated that KDM4B expression, lymph node metastasis (N stage), distant metastasis (M stage) and clinical stage were significantly associate with patient survival (Table 2). Furthermore, a multivariate Cox regression analysis further confirmed KDM4B expression, lymph node metastasis (N stage) and distant metastasis (M stage) as independent predictors of shorter OS (Table 2). More importantly, Kaplan-Meier analysis showed high KDM4B expression was significantly correlated with shorter survival (Log-rank, *p* = 0.028, Fig. 6d). Analysis of the gene expression with survival data from the TCGA database also showed similar results (Log-rank, *p* = 0.0301, Fig. 6e). Taken together, all these findings suggested that high expression of KDM4B indicated unfavorable prognosis.

**Table 1:** Correlation between KDM4B expression levels and clinicopathological parameters in 100 cases of colon canser

Variable	N	High KDM4B	Low KDM4B	*P* Value
Gender				
Male	59	39	20	
Female	41	28	13	> 0.9999
Age				
< 60	24	18	6	
≥ 60	76	49	27	0.4567
Tumor size(cm)				
< 5	34	24	10	
≥ 5	66	43	13	0.6574
T-stage				
1/2	3	0	3	
3/4	97	67	30	0.0337
N-stage				
N0	53	32	21	
N1	35	24	11	
N2	12	11	1	0.1112
M-stage				
M0	95	63	32	
M1	5	4	1	> 0.9999
Clinical Stage				
I/II	52	29	23	
III/IV	48	38	10	0.0188

**Table 2:** Univariable and multivariable analyses of overall survival (OS) and clinicopathologic variables in 100 cases of colon cancer

Variable	No.	HR (95% CI)	*p* value	HR (95% CI)	*p* value
Gender					
Male	59	1.262 (0.721–2.207)	0.415	NA	
Female	41				
Age					
< 60	24	1.449 (0.727–2.889)	0.292	NA	
≥ 60	76				
Tumor size(cm)					
< 5	34	1.43 (0.801–2.554)	0.226	NA	
≥ 5	66				
T-stage					
1/2	3	1.262 (0.761–2.092)	0.367	NA	
3/4	97				
N-stage					
N0	53	1.819 (1.219–2.712)	0.003	2.438 (1.258–4.724)	0.008
N1	35				
N2	12				
M-stage					
M0	95	5.142 (1.984–13.326)	0.001	9.299 (2.143–40.355)	0.003
M1	5				
Clinical Stage					
I/II	52	1.893 (1.206–2.973)	0.006	0.568 (0.249–1.297)	0.179
III/IV	48				
KDM4B expression					
Low	33	2.025 (1.062–3.863)	0.032	1.862 (0.964–3.597)	
High	67				0.044

## Discussion

In this study, we wished to elucidate the specific role of lysine demethylase KDM4B on CRC growth and glucose metabolism. Our findings suggest that KDM4B facilitates CRC growth and glucose metabolism by stimulating AKT activation. KDM4B can interact with TRAF6 and promote TRAF6-mediated the K63 chain ubiquitination of AKT which contributes to the enhancement of AKT membrane localization and phosphorylation.

The oncogenic activities of KDM4B have been extensively investigated in multiple cancers, including breast, prostate, bladder, ovarian, gastric and colorectal cancer. KDM4B can promote tumor growth, apoptosis, metastasis and autophagy [9–12, 32]. All these functions cannot be separated from the support of energy metabolism. Compared to normal cells, cancer cells rewire their metabolism to differently utilize glucose for their energy needs. In cancer cells, glucose is converted to energy primarily by aerobic glycolysis [33]. This faster conversion of glucose to energy is required to meet the needs of rapidly growing cancer cells.

Recently, KDM3A, a demethylase that removes methyl form histone lysine H3K9, has been reported to promote urinary bladder cancer progression by enhancing glycolysis through coactivation of hypoxia inducible factor 1α [34]. KDM5B, another demethylase, has been reported to participate in the regulation of islet function and glucose homeostasis [35]. We also notice that the loss of KDM4B has been reported to result in metabolic dysfunction [17]. Consider the oncogenic role of KDM4B in multiple cancers, we wonder whether KDM4B can participate in glucose metabolism and the mechanism should be further investigated. In the present study, we identify a role for KDMB in promoting glucose uptake from extracellular environment by stimulating TRAF6-mediated AKT activation.

In previous studies, the role of KDM4 was mostly focused on the histone posttranslational modifications that regulate chromatin structure in nucleus for the past studies. The posttranslational modifications include the demethylation of key lysine residues on the N-terminal tail of histone H3 such as H3K9me3, H3K27me3 and H3K36me3, which are involved in promoter silencing and activating transcription [8]. But it is still unclear whether the KDM4B has some role in non-histone modification. In the meantime, some studies have previously demonstrated that KDM4B is also localized in cytoplasm [36]. The role of KDM4B in cytoplasm is also need our investigate. In our study, we demonstrate that the intracellular localization of KDM4B is both in cytoplasm and nucleus by immunofluorescence analysis and the KDM4B in cytoplasm could interact with TRAF6 and promote TRAF6-mediated AKT activation (Fig. 3). These results broaden our understanding the role of KDM4B in cell signaling.

**Fig. 7:**
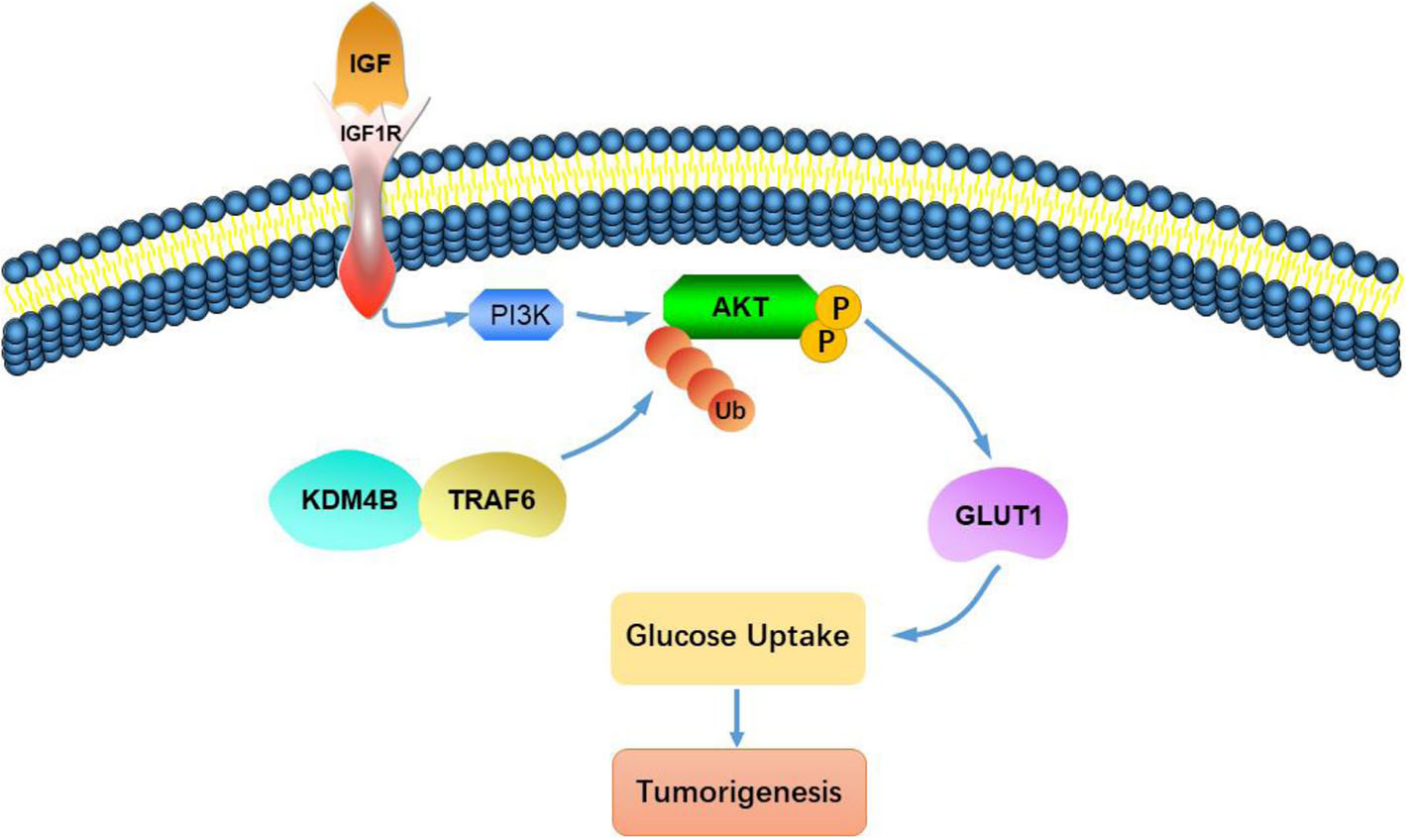
The proposed working model of KDM4B in regulating the GLUT1 expression by the activation of AKT

Glucose transport is located at a “gate” position in the glycolytic flux to respond to various stimuli crucial for the Warburg effect, which is directly mediated by GLUT proteins. Our results identify that KDMB plays an important role in glucose uptake and regulates the expression of GLUT1, the main glucose transporter in CRC cells. The result is consisted with the previous study. In LN Fu et al work, they reported that knockdown of KDM4B could transcriptionally inhibit GLUT1 expression through increasing H3K9 tri-methylation levels on GLUT1 promoter [37]. In our study, we present a novel mechanism which KDM4B participate in glucose metabolism by the regulation of GLUT1. Our study shows that KDM4B can interact with TRAF6 in cytoplasm and promote TRAF6-mediated K63-linked ubiquitination of AKT. The ubiquitination of AKT is required for activation of AKT and plays an important role in the regulation of GLUT1 expression (Fig. 7). This novel mechanism enables us to fully understand the important regulatory role of KDM4B in glucose metabolism.

PI3K/AKT signal pathway plays the central role in energy metabolism and coordinates diverse biological processes ranging from cell growth and differentiation to carcinogenesis [38, 39]. Our experiments reveal that KDM4B can acts closely upstream of or directly at AKT phosphorylation (Fig. 2). The membrane translocation of AKT from the cytosol to the membrane is the initial and essential step for AKT activation [40]. Our results indicate that the knockdown of KDM4B inhibits AKT activation through impairing AKT membrane recruitment (Fig. 3). While PI(3,4,5)P3 formation induced by PI3K is clearly critical for membrane recruitment and activation of AKT upon growth factor stimulation, recent studies have revealed that K63-linked ubiquitination of AKT induced by growth factor is also a prerequisite for these processes [41]. The ubiquitin E3 ligase TRAF6 promotes the K63-linked ubiquitination of AKT and is required for plasma membrane translocation of AKT [31]. In our study, we have found that knockdown of KDM4B impaired the K63-linked ubiquitination and KDM4B interacted with TRAF6 upon IGF-1 stimulation (Fig. 3).

Much more attention has been focused on the role of lysine methylase and de methylase on non-histone proteins. In GH Wang et al work, they identified SETDB1, a lysine methylation, as an AKT-interacting protein that methylates AKT at K64 to elicit AKT ubiquitination. SETDB1-mediated K64 methylation of AKT serves as a scaffold to recruit histone demethylase KDM4A, which brings AKT’s E3 ligases (TRAF6 and Skp2-SCF) to the AKT complex, thereby promoting AKT K63-linked ubiquitination, cell membrane recruitment and activation as well as tumorigenesis [42]. In M Dasgupta et al work, they reported STAT3-driven transcription depends upon the demethylation of K49 by EZH2 [43]. Our study also showed KDM4B could interact with TRAF6 in cytoplasm and promote TRAF6-mediated K63-linked ubiquitination of AKT. The role of KDM4B on non-histone proteins should be more investigated in our further study.

Studies aiming to develop effective diagnostic and prognostic biomarker for cancer are urgently needed. In this study, we found that KDM4B is frequently upregulated in colorectal cancer. Our findings strongly suggest that KDM4B may be a useful diagnostic biomarker for colorectal cancer. More importantly, higher KDM4B expression in primary tumors is significantly correlated with unfavorable tumor stages and shorter survival in patients with colorectal cancer (Fig. 6). There findings therefore underpin an oncogenic role for KDM4B in colorectal cancer. The reason why KDM4B is overexpressed in colorectal cancer remains to be explored.

## Conclusions

In summary, our study demonstrates that KDM4B facilitates colorectal cancer growth and glucose metabolism by stimulating TRAF6-mediated AKT activation, implicating that KDM4B is a potential molecular target for colorectal cancer treatment.

## Supplementary information

**Additional file 1 **: **Figure S1.** A BrdUrd incorporation into DNA and DNA content in nuclei were determined by flow cytometry analysis in KDM4B-overexpressed LoVo cells. B Intracellular glucose uptake was evaluated by 2-NBDG, a fluorescently tagged glucose derivative in KDM4B-depressed SW620 cells (siKDM4B 1#/2#) and KDM4B-overexpressed SW620 cells (KDM4B). C GSE9348 RNA sequencing from GEO database was performed in CRC tissues and normal tissues. Pathway enrichment analysis revealed that Glucose transport pathway was more correlated in CRC tissues. **Figure S2.** A The expression of GLUT1 and GLUT2 in KDM4B-depressed LoVo cells in mRNA levels. B and C The expression of GLUT1 was detected in KDM4B-depressed LoVo/SW620 cells transfected with siControl and siKDM4B 1#/2#. D and E The expression of GLUT1 was detected in KDM4B-overexpressed LoVo/HCT116 cells transfected with KDM4B plasmid. **Figure S3.** A The phosphorylation of AKT at Thr308 and Ser 473 and the expression of GLUT1 was detected in KDM4B-depressed HCT116 cells. B The membrane fractions, cytoplasm fractions and whole cell extracts were collected in KDM4B-depressed SW620 cells to measure the phosphorylation of AKT at Thr308 and Ser 473. C BrdUrd incorporation into DNA and DNA content in nuclei were determined by flow cytometry analysis in KDM4B-overexpressed LoVo cells treated with/without LY294002. D Intracellular glucose uptake was evaluated by 2-NBDG in KDM4B-overexpressed LoVo cells treated with/without LY294002.

## Data Availability

All of the data and material in this paper are available when requested.

## Abbreviations

BrdUrd: 5-bromo-2′-deoxyuridine
CRC: Colorectal cancer
GLUT1: Glucose transporter 1
IGF-1: Insulin like growth factor 1
myr-AKT: myristoylated AKT
PDK1: Pyruvate dehydrogenase kinase 1
TRAF6: TNF receptor associated factor 6
TXNIP: Thioredoxin interacting protein
