# Supplementary material for: KDM4B facilitates colorectal cancer growth and glucose metabolism by stimulating TRAF6-mediated AKT activation
Source: J Exp Clin Cancer Res. 2020 Jan 13;39:12. doi: 10.1186/s13046-020-1522-3 (PMC6958723; doi:10.1186/s13046-020-1522-3)
Supplement: Supplementary file 1 — Additional file 1 : Figure S1. A BrdUrd incorporation into DNA and DNA content in nuclei were determined by flow cytometry analysis in KDM4B-overexpressed LoVo cells. B Intracellular glucose uptake was evaluated by 2-NBDG, a fluorescently tagged glucose derivative in KDM4B-depressed SW620 cells (siKDM4B 1#/2#) and KDM4B-overexpressed SW620 cells (KDM4B). C GSE9348 RNA sequencing from GEO database was performed in CRC tissues and normal tissues. Pathway enrichment analysis revealed that Glucose transport pathway was more correlated in CRC tissues. Figure S2. A The expression of GLUT1 and GLUT2 in KDM4B-depressed LoVo cells in mRNA levels. B and C The expression of GLUT1 was detected in KDM4B-depressed LoVo/SW620 cells transfected with siControl and siKDM4B 1#/2#. D and E The expression of GLUT1 was detected in KDM4B-overexpressed LoVo/HCT116 cells transfected with KDM4B plasmid. Figure S3. A The phosphorylation of AKT at Thr308 and Ser 473 and the expression of GLUT1 was detected in KDM4B-depressed HCT116 cells. B The membrane fractions, cytoplasm fractions and whole cell extracts were collected in KDM4B-depressed SW620 cells to measure the phosphorylation of AKT at Thr308 and Ser 473. C BrdUrd incorporation into DNA and DNA content in nuclei were determined by flow cytometry analysis in KDM4B-overexpressed LoVo cells treated with/without LY294002. D Intracellular glucose uptake was evaluated by 2-NBDG in KDM4B-overexpressed LoVo cells treated with/without LY294002. [file 13046_2020_1522_MOESM1_ESM.pptx]

## Slide 1
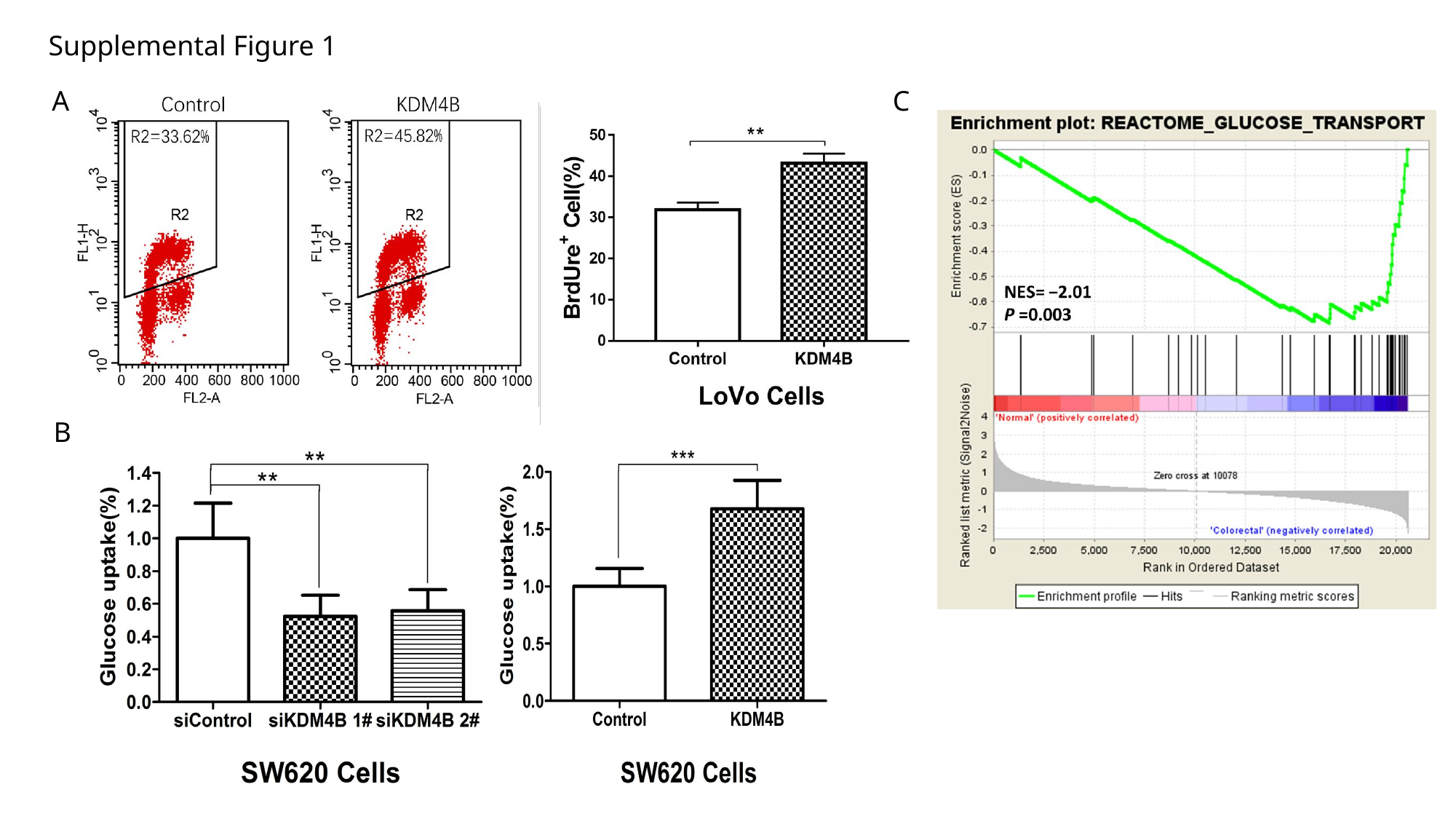

Supplemental Figure 1
A
C
B

## Slide 2
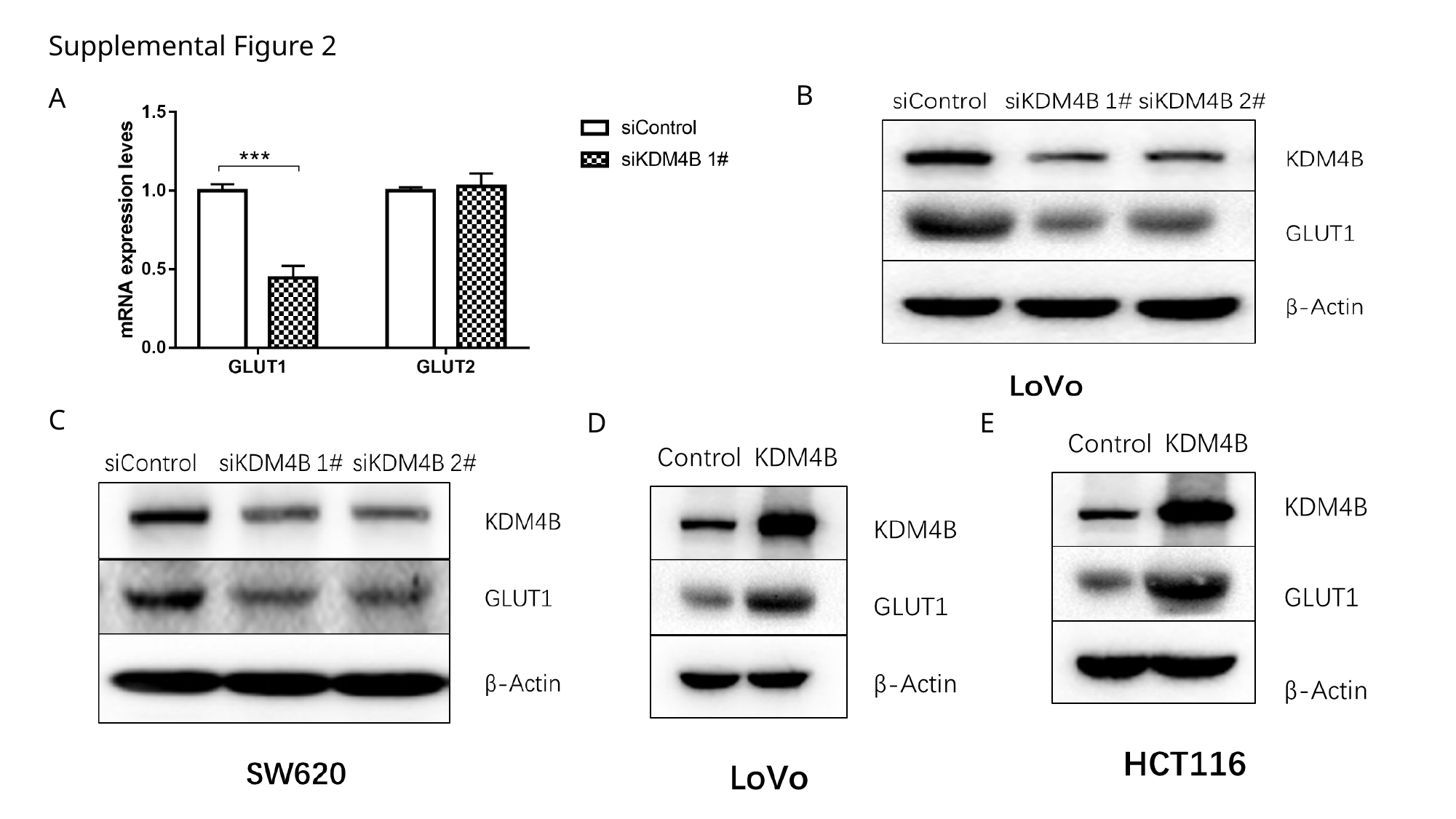

Supplemental Figure 2
B
A
C
E
D

## Slide 3
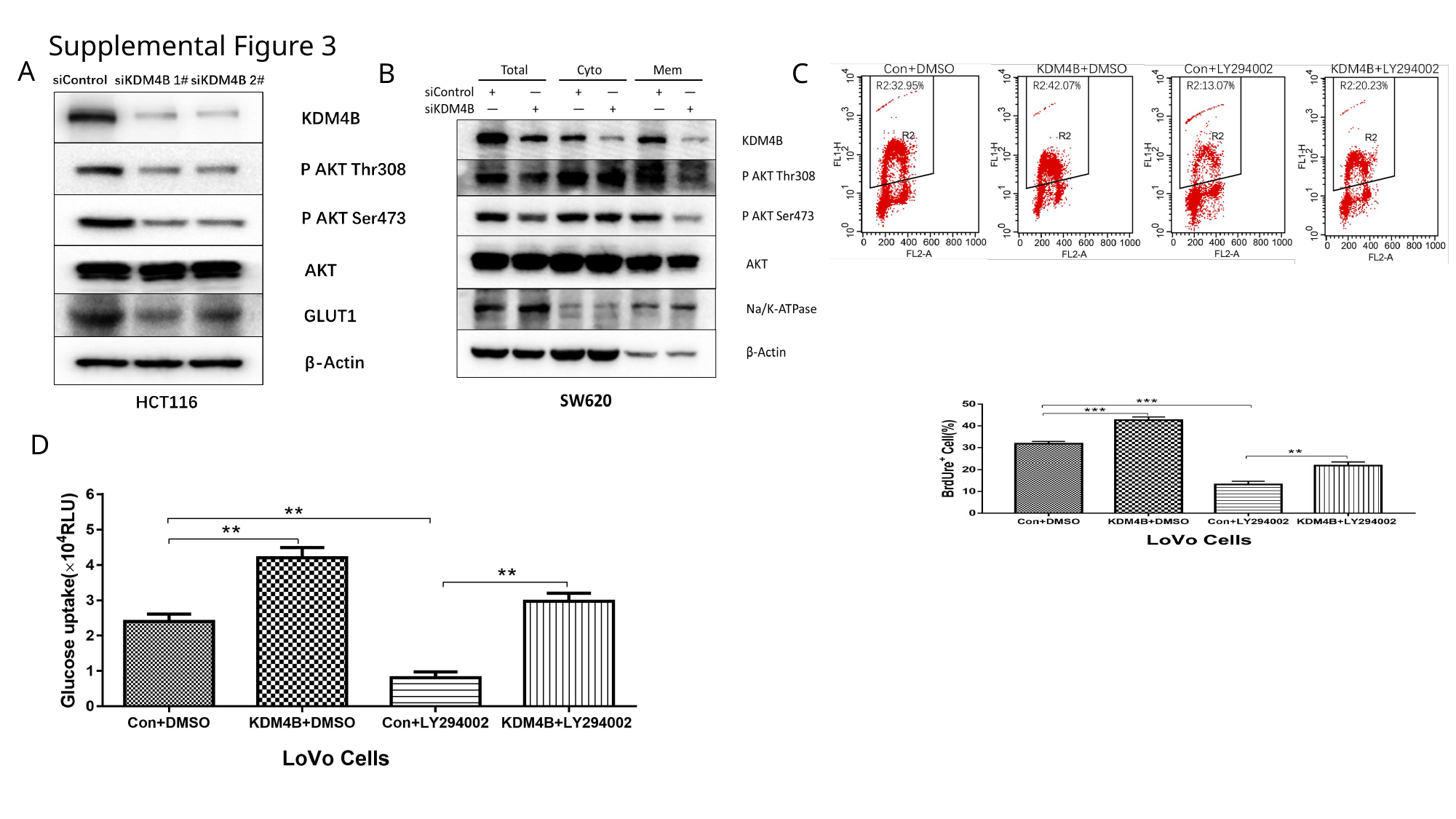

Supplemental Figure 3
A
B
C
D
